# Supplementary material for: The genome sequence of the Sycamore Piercer, Pammene aurita (Razowski, 1991)
Source: Wellcome Open Res. 2023 Apr 12;8:160. [Version 1] doi: 10.12688/wellcomeopenres.19243.1 (PMC11101136; doi:10.12688/wellcomeopenres.19243.1)
Supplement: Peer review history

# Reviewer report: Reviewer response for version 1

**DOI:** 10.21956/wellcomeopenres.21326.r78464

**Published:** 2024-05-16

**Authors:** Yash Sondhi

**Affiliations:** 1 University of Florida, Florida, USA

Overview:

 The report sequences the genome of the SYcaoTortricidae moth *Pammene aurita* (Sycamore piercer) using long read sequencing. They use Hi-C data to build the chromosome scaffold map is high quality and clearly identifies 28 chromosomes. They also assemble a mitochondrial genome and provide some future plans for an ensemble based gene prediction and annotation. As expected from Pac-Bio data, the assembly has a high N5- of 37.5 M and is 1041.8 Mb. There is the primary and secondary assembly representing some version of the phased assemblies are deposited. 

 The authors do a good job describing the life history and biogeography of the moth and report the methods and tools they use to generate and assess the genome well. 

 Minor comments

 1)Could the authors briefly discuss how the results of 1000 MB  chromosome and 28 chromosomes match with any other previous reports of closely related species? 

 2)The authors mention Ensemble prediction will be done from the remaining RNA-seq data. Can details of the RNA-sequencing be mentioned, are they whole animal or specific tissues and are the raw RNA reads accessible? Having access to this data will be valuable for confirming the gene prediction models if this data is available.

Are sufficient details of methods and materials provided to allow replication by others?

Yes

Is the rationale for creating the dataset(s) clearly described?

Yes

Are the datasets clearly presented in a useable and accessible format?

Yes

Are the protocols appropriate and is the work technically sound?

Yes

Reviewer Expertise:

Lepdiotera molecular evlution, sensory biology, sytematics and taxonomy

I confirm that I have read this submission and believe that I have an appropriate level of expertise to confirm that it is of an acceptable scientific standard.

# Reviewer report: Reviewer response for version 1

**DOI:** 10.21956/wellcomeopenres.21326.r70859

**Published:** 2024-05-13

**Authors:** Niklas Wahlberg

**Affiliations:** 1 Department of Biology, Lund university, Lund, Sweden

This report on the genome of Pammene aurita (Lepidoptera: Tortricidae) follows the standard format of genome reports, and as far as I can see, all looks good. It is interesting that the genome size is about double that of the average lepidopteran genome, and eventually it will be interesting to do a comparison of tortricid genomes to see how genome size evolves over time.

Are sufficient details of methods and materials provided to allow replication by others?

Yes

Is the rationale for creating the dataset(s) clearly described?

Yes

Are the datasets clearly presented in a useable and accessible format?

Yes

Are the protocols appropriate and is the work technically sound?

Yes

Reviewer Expertise:

Phylogenomics, systematics, evolution

I confirm that I have read this submission and believe that I have an appropriate level of expertise to confirm that it is of an acceptable scientific standard.

# Reviewer report: Reviewer response for version 1

**DOI:** 10.21956/wellcomeopenres.21326.r78465

**Published:** 2024-04-30

**Authors:** Panagiotis Ioannidis

**Affiliations:** 1 Foundation for Research & Technology Hellas, Crete, Greece

This genome report describes the sequencing and assembly of a tortricid

 moth (Lepidoptera). The authors provide sufficient background in the

 Introduction, in relation to the life cycle and geographical range of

 the animal. Additionally, the methods describe sufficiently the

 experimental procedures so that others can replicate their analysis.

 The resulting assembly is excellent (as expected for HiFi reads + HiC),

 both in terms of genome contiguity, as well as in gene content (BUSCO).

 Thus, I believe that it is suitable for use in downstream analyses (e.g.

 gene prediction, comparative genomics and phylogenomics).

 I have some comments of minor importance for the authors:

 1) Even though the authors have provided enough details about the life

 cycle and geographical range, I would expect to see a few more things

 about this lepidopteran's ecology (e.g. is it a pest?). Additionally, a

 bit more background about the genomic resources (genome/transcriptome

 data) that have already been generated by others, would also be a nice

 thing to report in the Introduction. I believe that both points (ecology

 and already available genomic data) will emphasize the importance of the

 herein published genome assembly.

 2) I saw the statement about gene prediction in the "Data availability"

 section and was wondering if the RNAseq data are from the same

 species/population/individuals that were used for genome sequencing. In

 general, it would probably be better if the authors also provided a

 predicted gene set for anyone interested in quickly performing any

 comparative analyses (because I'm guessing that gene prediction via

 Ensembl will take some time). But I also understand that such a task is

 not trivial and maybe it's not even within the scope of these genome

 reports.

 3) Also, regarding the assembly size (~1.0 Gbp), do the authors know if

 it is close to the expected genome size for this animal? If there are no

 clues about its genome size from other studies, do the authors know what

 is the genome size of other, related Lepidoptera?

 4) Similarly to the above comment, do the authors know if the total

 number of chromosomes (27 + 1) identified for this animal makes sense

 compared to what is known for this species, or for related species.

Are sufficient details of methods and materials provided to allow replication by others?

Yes

Is the rationale for creating the dataset(s) clearly described?

Yes

Are the datasets clearly presented in a useable and accessible format?

Yes

Are the protocols appropriate and is the work technically sound?

Yes

Reviewer Expertise:

insect genomics and transcriptomics, bioinformatics

I confirm that I have read this submission and believe that I have an appropriate level of expertise to confirm that it is of an acceptable scientific standard.
